# Poor prognostic factors in patients with newly diagnosed intestinal Adamantiades-Behçet’s disease in the Shanghai Adamantiades-Behçet’s disease database: a prospective cohort study

**DOI:** 10.1186/s13023-019-1228-9

**Published:** 2019-11-28

**Authors:** Liang Zhang, Yun Tian, Jing-Fen Ye, Chen-Hong Lin, Jian-Long Guan

**Affiliations:** Department of RHEUMATOLOGY and Immunology, Huadong Hospital affiliated to Fudan University, #221 yan’an west Road, Shanghai, 200040 People’s Republic of China

**Keywords:** Adamantiades-Behçet’s disease, Intestinal ulcers, Prognostic factors, Recurrence

## Abstract

**Background:**

Adamantiades-Behçet’s Disease (ABD) is an immunological recurrent systemic vasculitis with a chronic course. We investigated the predictors of long-term flare-ups, poor outcomes and event-free survival in Chinese non-surgical patients with intestinal ABD.

**Methods:**

This was a prospective cohort study of 109 intestinal ABD patients seen in our institution between October 2012 and January 2019 who met the international criteria for ABD and had intestinal ulcers confirmed on colonoscopy. Predictors of relapses and poor outcomes, event-free survival were calculated using logistic regression models and Cox proportional hazard regression models, respectively.

**Results:**

Sixty-six intestinal ABD patients (60.55%) had ileocecal ulcers; 19 patients (17.43%) presented with colorectum ulcers; 24 patients (22.02%) showed both ileocecal and colorectum ulcers. 7 patients (6.42%) experienced at least 1 flare-up of intestinal ulcers. 38 patients (34.86%) complained of non-healing intestinal ulcers. In multivariate analysis, location of intestinal ulcers (ileocecal and colorectum) (odd ratio (OR) 7.498 [95% confidence interval [95% CI] 1.844–30.480]), erythrocyte sedimentation rate (ESR) > 24 mm/h (OR 5.966 [95% CI 1.734–20.528]), treatment with infliximab (IFX) (OR 0.130 [95% CI 0.024–0.715]), and poor compliance (OR 11.730 [95% CI 2.341–58.781]) were independently correlated with a poor outcome. After a median follow-up of 28 months, 45 intestinal ABD patients (41.28%) underwent adverse events. Factors independently associated with shorter event-free survival were early onset of ABD (< 7 years) (hazard ratio (HR) 2.431 [95% CI 1.240–4.764]) and poor compliance (HR 3.058 [95% CI 1.612–5.800]).

**Conclusion:**

Distribution of intestinal ulcers (ileocecal and colorectum), ESR > 24 mm/h, treatment without IFX, and poor compliance were independent risk factors for poor outcomes in non-surgical intestinal ABD patients.

## Background

Adamantiades-Behçet’s Disease (ABD) is a chronic inflammatory autoimmune disorder with unknown pathogenesis, characterized by recurrent oral and genital ulcers, skin lesions, uveitis, arthritis and intestinal, cardiovascular, and neurological involvement [1–3]. Intestinal Adamantiades-Behçet’s Disease (ABD) is diagnosed by the presence of intestinal ulcers, the features of which include typical intestinal ulcers (isolated, round/oval and deep ulcers with discrete margins in the ileocecal area) and atypical ulcers (multiple, volcano or geographic ulcers in other lower gastrointestinal areas), and systemic manifestations fulfilling the criteria of International Study Group (ISG) for ABD [4–6].

Intestinal involvement occurs in 10–20% of patients [7]. Intestinal ABD has cumulative relapse rates or 25 and 45% at 2 and 5 years, respectively [8]. The intestinal ulcers of intestinal ABD are mostly located in the terminal ileum and the cecum, and the most common intestinal symptom is abdominal pain, ranging from mild to severe, with or without fever, diarrhea, hematochezia, or weight loss [5, 8, 9]. intestinal ABD patients may experience such complications as intestinal bleeding, perforation, fistula and obstruction. Massive intestinal bleeding or acute intestinal perforation might be life-threatening and could substantially increase mortality [9–11]. There are reported relationships between elevated inflammatory indexes (including erythrocyte sedimentation rate (ESR) and C-reactive protein (CRP) and disease activity of intestinal ABD [12–14]. Patient compliance might also be an important determinant of disease outcomes. High proportions of poor compliance in rheumatic diseases varied from 20 to 90%, directly or indirectly leading to severe consequences [15, 16].

Despite the fact that clinical, colonoscopic features and outcomes of surgery and early readmission have been extensively identified, there have been few studies of long-term outcomes of non-surgical intestinal ABD patients in the Chinese population [17–19]. Therefore, the propose of our study was to investigate the risk factors for relapses and poor outcomes in Chinese non-surgical intestinal ABD patients.

## Methods

### Patients

We prospectively enrolled all followed-up patients who had been treated in the Department of RHEUMATOLOGY and Immunology of Huadong Hospital affiliated with Fudan University, Shanghai, China between October 2012 and January 2019. Of a cohort of 1115 ABD patients, 109 (9.78%) were newly diagnosed with non-surgical intestinal ABD. All 109 patients fulfilled the criteria of International Study Group for ABD [4]. The diagnosis of intestinal ABD was confirmed by identifying intestinal ulcers on colonoscopy that were not explained by any other intestinal diseases. Patients were excluded if they had upper gastrointestinal ulcers (including esophageal and gastric ulcers).

### Data collection and outcome assessment

The following information was collected: gender, age of ABD onset, duration of ABD, clinical manifestations of ABD (oral ulcer, genital ulceration, skin lesions and ocular, vascular, neurological and blood involvement), intestinal symptoms, colonoscopy features (distribution of intestinal ulcers, size and number), laboratory indexes (white blood cells (WBC), hemoglobin (Hb), platelets (PLT), ESR, CRP, fecal occult blood test (FTOB), tuberculosis (TB) infection T cell spot test (T-SPOT.TB) and hepatitis B virus DNA (HBV-DNA)), treatment, and patient compliance. Intestinal symptoms included abdominal pain, diarrhea, hematochezia, and fever. The distribution of intestinal ulcers was divided into ileocecal ulcers alone, colorectum ulcers alone, and both ileocecal and colorectum ulcers. Treatment in intestinal ABD patients included conventional drugs (steroids and immunosuppressants) and biologics (infliximab (IFX) and etanercept). Poor compliance on the part of intestinal ABD patients was defined as patients who could not properly follow the recommendations provided by rheumatologists. Relapses of intestinal ABD were defined as recurrences of intestinal ulcers on repeated enteroscopy after ulcer healing. Poor outcome of intestinal ABD was defined as repeated colonoscopy showing intestinal ulcers after standard treatment modification or intensification. An event was defined as the appearance of relapse of intestinal ABD or non-healing intestinal ulcers during a follow-up period.

### Statistical analysis

Data were described as the number (%) or median (25–75% interquartile range [IQR]) for categorical and quantitative variables, respectively. Factors associated with poor outcome of intestinal ABD (non-healing intestinal ulcers) were subjected to univariate analysis using Wilcoxon’s test and χ^2^/Fisher’s exact tests for quantitative and categorical variables, respectively. The predictive factors of non-healing ulcers that had *P*-values of < 0.20 in univariate analysis were included in a multiple logistic regression model. Variables were selected by a backward stepwise procedure based on the *P*-value.

Factors correlated with relapses or non-healing intestinal ulcers were subjected to univariate analysis using the log rank test and were expressed as hazard ratio (HR) and 95% confidence interval (95% CI). All factors with *P*-values less than 0.20 were assessed using a multiple Cox model. Variable selection was conducted using a backward stepwise procedure grounded on *P*-value.

All tests were two-sided with 0.05 significance level. Analyses were performed using SPSS 22.0.

## Results

Baseline characteristics. The basic clinical features of the 109 non-surgical intestinal ABD patients are presented in Table 1.

**Table 1:** Baseline characteristics of the 109 newly diagnosed patients with IBS

	All newly diagnosed IBS patients	IBS with ileocecal ulcers	IBS with colorectum ulcers	IBS with both ileocecal and colorectum ulcers	*P*
N (%)	109	66 (60.55)	19 (17.43)	24 (22.02)	–
Follow-up, median (IQR) months	28.00 (14.00–46.00)	27.00 (12.00–47.50)	29.00 (11.00–46.00)	29.00 (20.25–42.75)	–
Male Sex, no. (%)	57 (52.29)	31 (46.97)	11 (57.89)	15 (62.50)	0.370
Age at the diagnosis of BS, median (IQR) years	35.00 (25.00–50.00)	33.00 (25.00–48.75)	34.00 (19.00–48.00)	46.50 (28.25–52.25)	0.028
Onset of BS, median (IQR) years	7.00 (3.00–10.00)	7.50 (4.00–10.00)	7.00 (2.00–18.00)	6.50 (2.25–10.75)	0.780
Oral ulceration, no. (%)	106 (92.25)	64 (96.97)	18 (94.74)	24 (100.00)	0.564
Genital ulcer, no. (%)	75 (68.81)	47 (71.21)	13 (68.42)	15 (62.50)	0.732
Skin manifestations, no. (%)					
erythema nodosum	28 (25.69)	15 (22.73)	7 (36.84)	6 (25.00)	0.461
epifolliculitis	30 (27.52)	20 (30.30)	5 (26.32)	5 (20.83)	0.668
Positive pathergy reaction	3 (2.75)	3 (4.55)	0 (0)	0 (0)	–
Systemic involvement, no. (%)					
ocular involvement	8 (7.34)	5 (7.58)	1 (5.26)	2 (8.33)	0.923
vascular involvement	6 (5.50)	4 (6.06)	0 (0)	2 (8.33)	–
neurological involvement	3 (2.75)	1 (1.52)	1 (5.26)	1 (4.17)	0.605
blood involvement	5 (4.59)	3 (4.55)	0 (0)	2 (8.33)	–
Intestinal symptoms	61 (55.96)	32 (48.48)	13 (68.42)	16 (66.67)	0.149
Endoscopic characteristics, no. (%)					
Number, ≥ 3	62 (56.88)	22 (33.33)	13 (68.42)	20 (83.33)	0.000
Size, > 1 cm	33 (30.28)	20 (30.30)	4 (21.05)	9 (37.50)	0.507
Lab parameters, no. (%)					
WBC, × 10^9^/L	6.40 (5.15–9.20)	6.20 (5.28–9.33)	6.40 (4.70–8.10)	7.10 (4.73–12.98)	0.529
Hb, g/L	123.00 (107.00–136.00)	124.50 (111.25–135.00)	126.00 (105.00–138.00)	114.00 (102.00–138.75)	0.160
PLT, × 10^9^/L	230.00 (185.50–299.50)	220.00 (184.00–286.50)	263.00 (222.00–356.00)	249.00 (189.50–300.25)	0.762
ESR, mm/h	24.00 (10.00–43.50)	22.00 (10.00–40.25)	15.00 (6.00–40.00)	39.00 (20.50–67.75)	0.037
CRP, mg/L	10.000 (3.750–37.485)	10.000 (3.875–18.925)	10.00 (0.80–14.50)	21.50 (5.15–54.15)	0.116
Positive FOBT^+^	23 (21.10)	10 (15.15)	6 (31.58)	7 (29.17)	0.166
Positive T-SPOT	19 (17.43)	13 (19.70)	4 (21.05)	2 (8.33)	0.409
HBV-DNA	6 (5.50)	4 (6.06)	2 (10.53)	0 (0)	–

^+^FOBT: fecal occult blood test

Male patients are accounted for almost half in intestinal ABD. The median age at diagnosis of intestinal ABD patients was 35 years (interquartile range (IQR) 25–50 years). The median onset of ABD was 7 years (IQR 3–10 years). Of these, 106 patients (92.25%) had oral aphthous ulcers; 75 patients (68.81%) had genital ulceration; skin lesions including erythema nodosum (25.69%), epifolliculitis (27.52%), impetigo (0.92%), and positive pathergy reaction (2.75%). Considering the coexistence of other organ lesions, 8 patients (7.34%) presented with ocular lesions; 6 patients (5.50%) showed vascular involvement; 3 patients (2.75%) complained of central nervous system (CNS) involvement; and 5 patients (4.59%) had blood system manifestations. The intestinal symptoms were observed in 61 of 109 intestinal ABD patients (55.96%). The number (≥ 3) and size (> 1 cm) of intestinal ulcers were found in 62 patients (56.88%) and 33 patients (30.28%), respectively.

Description of intestinal ABD according to distribution of intestinal ulcers. As demonstrated in Table 1, the distribution of intestinal ulcers in intestinal ABD patients included ileocecal ulcers (60.55%), colorectum ulcers (17.43%), and both ileocecal and colorectum ulcers (22.02%). There was a higher prevalence of earlier onset in intestinal ABD patients only located in ileocecal ulcers compared to the other 2 groups (*P* = 0.028). There were no significant difference in the sex and durations of ABD onset among the 3 groups. The principle symptoms of ABD (oral ulceration, genital ulcer, skin lesions, uveitis, and neurological involvement) were similar in the 3 groups. The intestinal symptoms and size of intestinal ulcers (> 1 cm) were similar among the 3 groups, but the number of intestinal ulcers (≥ 3) was more frequent in group with both ileocecal and colorectum ulcers (*P* = 0.000). The median ESR level (39.00 mm/h [IQR 20.50–67.75]) was significantly higher in the intestinal ABD patients with both ileocecal and colorectum ulcers (*P* = 0.037). A trend toward lower Hb levels and higher CRP levels were seen in the group with both ileocecal and colorectum ulcers (*P* = 0.160 and 0.116, respectively).

Factors associated with the risk of intestinal ulcers flare-up. During a median follow-up period of 21 months (IQR 9–48 months), 7 of the 109 patients (6.42%) experienced at least 1 flare-up of intestinal ulcers. In univariate analysis, the factors related to relapses of intestinal ABD included symptomatic intestinal ABD, number of intestinal ulcers (≥ 3), and size of intestinal ulcers (> 1 cm) (Table 2).

**Table 2:** Factors associated with the risk of intestinal ulcers flare-ups

	Univariate analysis		Multivariate analysis	
	OR (95% CI)	*P*	OR (95% CI)	*P*
Male sex	2.28 (0.46–11.25)	0.441	–	–
Age at the diagnosis of BS > 35 years	2.64 (0.54–13.03)	0.262	–	–
Onset of BS (< 7 years)	1.31 (0.31–5.58)	1.000	–	–
Intestinal symptoms	4.72 (0.59–37.90)	0.132	5.14 (0.58–45.12)	0.140
Endoscopic characteristics				
Location (ileocecal and colorectum)	0.59 (0.08–4.67)	1.000	–	–
Number, ≥ 3	0.16 (0.02–1.31)	0.060	0.15 (0.02–1.29)	0.084
Size, > 1 cm	3.07 (0.73–12.96)	0.196	–	–
Lab parameters				
WBC > 6.4, × 10^9^/L	0.76 (0.18–3.25)	1.000	–	–
Hb < 123, g/L	0.79 (0.19–3.38)	1.000	–	–
ESR > 24, mm/h	1.36 (0.32–5.78)	0.716	–	–
CRP > 10, mg/L	2.55 (0.52–12.57)	0.271	–	–
Positive FOBT^+^	1.50 (0.31–7.22)	0.617	–	–
Positive T-SPOT	0.79 (0.10–6.18)	1.000	–	–
HBV-DNA	6.87 (1.66–28.36)	0.287	–	–
Treatment				
Biologics	1.83 (0.37–9.00)	0.697	–	–
IFX therapy^#^	1.41 (0.33–6.00)	0.711	–	–
Poor compliance	0.38 (0.05–3.06)	0.673	–	–

^+^FOBT: fecal occult blood test

^#^IFX therapy was administrated at 0,2, and 6 weeks (intravenous), and then maintained with the same dosage of IFX every 8 weeks

**Table 3:** Factors associated with the risk of poor outcome (non-healing ulcers)

	Univariate analysis		Multivariate analysis	
	OR (95% CI)	*P*	OR (95% CI)	*P*
Male sex	1.01 (0.61–1.70)	0.959	–	–
Age at the diagnosis of BS > 35 years	1.06 (0.63–1.77)	0.833	–	–
Onset of BS < 7 years	1.39 (0.85–2.27)	0.190	–	–
Intestinal symptoms	1.08 (0.64–1.82)	0.766	–	–
Endoscopic characteristics			–	–
Location (ileocecal and colorectum)	2.58 (1.63–4.07)	0.000	7.10 (1.81–27.86)	0.005
Number, ≥ 3	1.21 (0.72–2.04)	0.463	–	–
Size, > 1 cm	1.68 (1.02–2.76)	0.049	3.15 (0.91–10.84)	0.070
Lab parameters				
WBC > 6.4, × 10^9^/L	1.56 (0.92–2.66)	0.093	–	–
Hb < 123, g/L	1.81 (1.05–3.11)	0.026	–	–
ESR > 24, mm/h	2.85 (1.54–5.28)	0.000	5.97 (1.73–20.53)	0.005
CRP > 44, mg/L	1.94 (1.19–3.17)	0.014	–	–
Positive FOBT^+^	1.16 (0.64–2.09)	0.629	–	–
Positive T-SPOT	0.56 (0.22–1.39)	0.164	0.25 (0.05–1.35)	0.107
HBV-DNA	0.95 (0.30–3.05)	1.000	–	–
Treatment			–	–
Biologics	0.30 (0.17–0.54)	0.000	–	–
IFX therapy^#^	0.24 (0.12–0.50)	0.000	0.18 (0.04–0.85)	0.031
Poor compliance	3.95 (2.35–6.62)	0.000	8.56 (1.91–38.26)	0.005

^+^FOBT: fecal occult blood test

^#^IFX therapy was administrated at 0,2, and 6 weeks (intravenous), and then maintained with the same dosage of IFX every 8 weeks

In multivariate analysis, there were no factors independently associated with ulcers flare-up. But a trend toward decreases the odds of relapse was found in number of intestinal ulcers (≥ 3) (OR 0.148 [95% CI 0.017–1.291]).

Factors associated with the poor outcome of intestinal ABD (non-healing intestinal ulcers). During follow-up, 38 patients complained of non-healing intestinal ulcers. We analyzed the factors correlated with the risk of intestinal ABD with poor outcomes (non-healing intestinal ulcers) (Table 3). In univariate analysis, the factors related to poor prognoses included location of intestinal ulcers (ileocecal and colorectum), size of intestinal ulcers (> 1 cm), abnormal blood parameters (WBC > 6.4 × 109/L, Hb < 123 g/L, ESR > 24 mm/h, CRP > 44 mg/L), and poor compliance. Biological agents, especially IFX, had protective impact on the prognoses of intestinal ABD patients. In multivariate analysis, location of intestinal ulcers (ileocecal and colorectum) (OR 7.100 [95% CI 1.810–27.855]), ESR > 24 mm/h (OR 5.966 [95% CI 1.734–20.528]), IFX therapy (OR 0.175 [95% CI 0.036–0.852]), and poor compliance (OR 8.557 [95% CI 1.914–38.255]) were independently correlated with a poor outcome. A trend toward more frequent poor outcomes were observed for size of intestinal ulcers (> 1 cm) (OR 3.198 [95% CI 0.901–11.350]) and positive T-SPOT (OR 0.250 [95% CI 0.046–1.351]).

Event-free survival of intestinal ABD is an event was considered to be the appearance of relapse or non-healing intestinal ulcer. Using a COX proportional hazards model, the factors that had negative impact on event-free survival were WBC > 6.4 × 109/L (HR 1.767 [95% CI 0.940–3.323]), Hb < 123 g/L (HR 1.552 [95% CI 0.826–2.916]), ESR > 24 mm/h (HR 2.176 [95% CI 1.107–4.276]), and poor compliance (HR 3.258 [95% CI 1.733–6.126]), but male sex (HR 0.571 [95% CI 0.299–1.092]), early onset of ABD (< 7 years) (HR 0.444 [95% CI 0.231–3.323]), and Biologics (HR 0.369 [95% CI 0.192–0.710]), IFX therapy (HR 0.461 [95% CI 0.224–0.948]) were associated with longer event-free survival by univariate analysis (Table 4).

**Table 4:** Factors associated with event-free survival of IBS

Risk factor	HR (95%CI)	*P*
Log Rank test risk factor		
Male sex	0.57 (0.30–1.09)	0.09
Onset of BS < 7 years	2.25 (1.17–4.33)	0.015
WBC > 6.4, × 10^9^/L	1.77 (0.94–3.32)	0.077
Hb < 123, g/L	1.55 (0.83–2.92)	0.172
ESR > 24, mm/h	2.18 (1.11–4.28)	0.024
Biologics	0.369 (0.192–0.710)	0.003
IFX therapy^#^	0.46 (0.22–0.95)	0.035
Poor compliance	3.26 (1.73–6.13)	0.000
Multiple Cox regression risk factor		
Onset of BS < 7 years	2.43 (1.24–4.76)	0.010
ESR > 24, mm/h	1.97 (0.99–3.91)	0.053
Poor compliance	3.06 (1.61–5.80)	0.001

^#^IFX therapy was administrated at 0, 2, and 6 weeks (intravenous), and then maintained with the same dosage of IFX every 8 weeks

In multivariate analysis, early onset of ABD (< 7 years) (HR 2.431 [95% CI 1.240–4.764]) and poor compliance (HR 3.058 [95% CI 1.612–5.800]) were independently correlated with shorter event-free survival. A trend toward shorter event-free survival was observed for ESR > 24 mm/h (HR 1.967 [95% CI 0.990–3.909]).

## Discussion

To the best of our knowledge, this is the first study to determine predictors of poor outcomes in Chinese non-surgical intestinal ABD patients. We studied 109 newly diagnosed non-surgical intestinal ABD patients and followed them up in a prospectively designed schedule. The most significant finding was that the distribution of intestinal ulcers (ileocecal and colorectum), ESR > 24 mm/h, and poor compliance increased the risk of non-healing ulcers by 7.100-fold, 5.966-fold and 8.557-fold, respectively; IFX therapy was a protective factor that accelerated ulcer healing; in intestinal ABD patients, onset of ABD < 7 years and poor compliance gave rise to shorter event-free survivals of 2.431-fold and 3.058-fold, respectively.

Our intestinal ABD patients with poor outcomes (non-healing intestinal ulcers) accounted for 34.86%, slightly lower than the rate of 58.3% reported in other studies of intestinal ABD patients [10, 13, 18]; no patients died during follow-up. Previously, the identified independent risk factors for poor prognosis in intestinal ABD were male gender, mucosal healing, volcano-shaped ulcers, larger size of intestinal ulcers (> 2 cm) and elevated CRP levels (≥ 44 mg/L )[10, 20–22].

We found that location of intestinal ulcers (ileocecal and colorectum) and poor compliance were strong independent prognostic factors for poor outcomes (non-healing intestinal ulcers) in intestinal ABD patients. To our knowledge, no previous series has assessed the effect of location of intestinal ulcers (ileocecal and colorectum) and poor compliance on non-healing intestinal ulcers in Chinese non-surgical intestinal ABD patients. Regarding the association between distribution of intestinal ulcer, however, some authors found no association between distributions of intestinal ulcers and poor prognoses in intestinal ABD patients. Compared to other patients in two other groups (located in ileocecal alone or colorectum alone), these patients tended to be older, have more intestinal ulcers, lower Hb concentrations, and higher ESR or CRP levels, all of which are adverse items for poor outcomes in intestinal ABD.

A total of 30.27% of the patients showed poor compliance with treatment regimens. Similar frequencies were reported in other studies of rheumatic diseases [16, 23, 24]. Logistic regression models and Cox proportional hazards regression analysis revealed that poor compliance gave a 8.557-fold and 3.058-fold increased risk for poor outcomes and worse event-free survival, respectively. Poor compliance is an extremely common and challenging problem in ABD, caused by chronic long-term courses, concerns regarding effectiveness, side-effects of medications, and financial burdens. Beliefs regarding the necessity of therapeutics and side-effects were significant predictors of poor compliance and adherence in ABD patients [15]. With respect to poor compliance, scheduled and standard medication therapy is essential for maintaining remission in intestinal ABD. Some reports revealed that excellent compliance was the most remarkable patient-related factor for treatment success [25]. A large-scale retrospective study over 15 years showed that poor compliance gave a 5.6-fold greater risk of poor prognosis [16]. A study from Ireland reported that patients with poor compliance complained of low mood, increasing the rates of ABD flare-ups [26]. These data suggest that physical and mental treatments are equally important throughout the disease course.

We also observed that ESR > 24 mm/h and IFX therapy were independently predictive of non-healing intestinal ulcers for intestinal ABD. The size of intestinal ulcers (> 1 cm), Hb < 123 g/L, and elevated inflammatory biomarker levels (CRP > 44 mg/L) were correlated with non-healing ulcers in univariate analysis but not in multivariate analysis. Many studies, including our previous studies, have shown associations between elevated ESR level and intestinal ABD activity [13, 27, 28]. Nevertheless, to date, there is no study has demonstrated that high-level ESR was predictive of intestinal ABD. In a study of patients with Crohn’s disease, those with elevated ESR (> 15 mm/h) levels had an 8-fold greater rate of flare-ups [29]. Our study indicated, for the first time, that elevated ESR (> 24 mm/h) might be a prognostic factor for poor prognoses in non-surgical intestinal ABD patients.

IFX is the first-line therapy for moderate-to-severe intestinal ABD. Several reports, including our previous study, confirmed the safety and efficacy of IFX in the short- and long-term follow-up in intestinal ABD patients [30–33]. IFX was thought to alter the natural course of intestinal ABD. Furthermore, it is believed that IFX is most beneficial during the early-stage intestinal ABD, that is, before appearance of extensive or severe intestinal lesions. However, a Korean study in intestinal ABD patients revealed no association between use of biologics and good prognosis based on gender and age group [5]. This discrepancy may be explained by the fact that the previous study defined long-term clinical outcomes as readmission and cumulative frequencies of surgical operation, both of which were differ from the supposed poor outcomes (non-healing ulcers) defined in our study.

We also speculate that early onset of ABD (< 7 years) is an important factor related to the occurrence of adverse events (relapse or non-healing intestinal ulcers) in our intestinal ABD patients. Some authors found that early-onset intestinal ABD patients presented with more severe clinical symptoms and poorer clinical outcomes because of stronger immune responses and higher frequency of volcano-shaped ulcers, also significantly associated with recurrences and poor prognosis in intestinal ABD [18, 34].

We acknowledge several limitations in the present study. First, the study was not population-based, but rather was hospital-based; therefore, referral and selection bias could not be excluded. Nevertheless, intestinal ABD patients in our hospital came from across the whole country in China, possibly minimizing such biases. Second, we were not able to evaluate the complications, because the complication rate was too low to be calculated. Third, we did not consider psychological conditions or healthcare costs, both of which might influence outcomes of intestinal ABD. Nevertheless, in an unpublished study, we found that low income, high hospitalization costs and long-term chronic clinical course could directly lead to depression, thought to be related to poor outcomes in ABD patients.

## Conclusions

The distribution of intestinal ulcers (ileocecal and colorectum), ESR > 24 mm/h and poor compliance were independent predictive risk factors for non-healing ulcers in intestinal ABD. IFX therapy may improve the prognosis of intestinal ABD. Onset of ABD < 7 years and poor compliance both negatively influence event-free survival. Therefore, IFX therapy and sustained improvement in intestinal ABD patient compliance could greatly accelerate intestinal ulcer healing and prolong event-free survival.

## Data Availability

The datasets used and analyzed during the current study are available from the corresponding author on reasonable request.
